# Brain Tissue Oxygen Levels as a Perspective Therapeutic Target in Traumatic Brain Injury. Retrospective Cohort Study

**DOI:** 10.2478/jccm-2023-0001

**Published:** 2023-02-08

**Authors:** Gal Roman, Ondrej Hrdy, Kamil Vrbica, Jan Hudec, Andrej Mrlian, Martin Smrcka

**Affiliations:** 1 Department of Anaesthesiology and Intensive Care Medicine, Masaryk University and University Hospital Brno, Brno, Czech Republic; 2 Department of Neurosurgery, Masaryk University and University Hospital Brno, Brno, Czech Republic

**Keywords:** traumatic brain injury, critical care, Glasgow Outcome Scale, brain tissue oxygen

## Abstract

**Introduction:**

Management of traumatic brain injury (TBI) requires a multidisciplinary approach and represents a significant challenge for both neurosurgeons and intensivists. The role of brain tissue oxygenation (PbtO2) monitoring and its impact on posttraumatic outcomes remains a controversial topic.

**Aim of the study:**

Our study aimed to evaluate the impact of PbtO2 monitoring on mortality, 30 days and 6 months neurological outcomes in patients with severe TBI compared with those resulting from standard intracranial pressure (ICP) monitoring.

**Material and methods:**

In this retrospective cohort study, we analysed the outcomes of 77 patients with severe TBI who met the inclusion criteria. These patients were divided into two groups, including 37 patients who were managed with ICP and PbtO2 monitoring protocols and 40 patients who were managed using ICP protocols alone.

**Results:**

There were no significant differences in demographic data between the two groups. We found no statistically significant differences in mortality or Glasgow Outcome Scale (GOS) scores one month after TBI. However, our results revealed that GOS scores at 6 months had improved significantly among patients managed with PbtO2; this finding was particularly notable for Glasgow Outcome Scale (GOS) scores of 4–5. Close monitoring and management of reductions in PbtO2, particularly by increasing the fraction of inspired oxygen, was associated with higher partial pressures of oxygen in this group.

**Conclusions:**

Monitoring of PbtO2 may facilitate the appropriate evaluation and treatment of low PbtO2 and represents a promising tool for the management of patients with severe TBI. Additional studies will be needed to confirm these findings.

## Introduction

Traumatic brain injury (TBI) is a significant public health burden, with 69 million cases reported annually throughout the world. The incidence of TBI is approximately 1,200 cases per 100,000 persons [1]. Management of severe TBI currently focuses on minimizing secondary brain injury, with particular attention centred on the identification and treatment of elevated intracranial pressure (ICP). As ICP-guided therapy and complex management of pressure elevations may serve to improve outcomes after TBI, this strategy has been included in the current Guidelines for the Management of Severe Brain Injury [2]. However, as secondary brain injury may develop despite normal ICP levels, other parameters might be monitored to enhance patient outcomes [3, 4]. Recently an attempt has been made to integrate multimodality monitoring (ICP, PbtO2, and auto regulatory status) into decision support algorithms [5].

One parameter of interest in this regard is brain tissue oxygenation (PbtO_2_). Neurons have only a limited capacity to survive without sufficient oxygen. Short periods of reduced PbtO_2_ can lead to secondary brain injury and ischemia even in the absence of an observed increase in ICP [6, 7]. Although studies that addressed this issue were assessed during the preparation of recent guidelines, the evaluations that were available at that time yielded conflicting results. Therefore, no recommendations regarding PbtO_2_ monitoring were included. However, several studies that focus on the role of PbtO_2_ monitoring in the management of TBI patients have been published since that time. In one trial, 50 patients were randomized for treatment with traditional ICP-guided therapy or PbtO_2_-guided management. The survival rate for the patients in the PbtO_2_-guided group increased significantly at 3 and 6 months after the acute injury [8]. In 2017, Okonkwo et al. [9] published the results of a randomized trial in which outcomes of TBI patients treated with an ICP protocol alone or with an ICP protocol together with PbtO_2_ monitoring were compared. The results of this study revealed that patients managed with the combined PbtO_2_ and ICP monitoring protocol experienced less brain tissue hypoxia, reduced mortality, and increased rates of good recovery compared to patients managed with the ICP protocol alone. However, Green et al. [10] conducted a retrospective study of 74 patients treated for severe TBI in which outcomes from a group of 37 patients who underwent ICP monitoring alone were compared to those of another 37 patients who were managed with both ICP-guided and PbtO_2_-guided monitoring. In this latter study, survival and functional outcomes did not differ between groups at the time of hospital discharge.

As the results of these more recent studies also remain conflicting, we felt that more research into this question was needed. The present study aimed to analyse the effects of PbtO_2_-guided management on the outcomes in patients who were admitted to our Level I Trauma Centre for treatment of severe TBI compared with management guided by monitoring of intracranial pressure solely. Results of this study may help to determine the role of PbtO_2_ in the effective management of TBI patients.

## Material and Methods

This retrospective study was approved by the Ethics Committee of University Hospital Brno. Informed consent was waived because of the retrospective design of the study (Ref. Nr. 09-170221/EK, date of approval February 17, 2021). All patients admitted to the Department of Anaesthesiology and Intensive Care Medicine and the Department of Neurosurgery of University Hospital Brno (Level 1 Trauma Centre) for treatment of severe TBI between January 2010 and December 2015 were screened for eligibility. Inclusion criteria were as follows: severe TBI with a Glasgow Coma Scale (GCS) score ≤ 8 at admission, monitoring performed using ICP-guided protocols alone or ICP and PbtO_2_ protocols, and age between 18 and 65 years. Patients were excluded if they presented with dilated and fixed pupils or died within the first 24 hours after admission.

Patients were divided into two groups. In one group (ICP) the management was guided by monitoring of ICP. In the second group (ICP+PbtO2) the management was guided by monitoring of ICP and monitoring of PbtO2 (Integra Licox® Brain Tissue Oxygenation Monitoring System, Integra LifeScience, Saint Priest, France).

## The ICP group

### Neuromonitoring

The neuromonitoring probes were placed at Kocher’s point via a simple trepanation procedure. Insertion of the monitoring device was performed within the first 24 hours after admission to the intensive care unit (ICU). There was no strict protocol for the insertion of these devices, although several specific rules were followed. For example, the probe was inserted in the non-dominant hemisphere in cases of diffuse injury. In cases involving focal injury, the probe/probes were inserted into the injured hemisphere; in cases involving bilateral focal injuries, the probe/probes were inserted in the more severely injured side. The final choice of monitoring device and site of insertion was made by the attending neurosurgeon. The procedure was performed at the bedside to maintain valid calibration of the devices. After insertion of the probes, the patients underwent computed tomography (CT) scans to verify their correct position and to identify any bleeding complications that may have resulted from the insertion procedure. The probes were removed if the patient’s clinical status improved or if necessary to prevent infectious complications. The data were monitored continuously and recorded hourly in the medical records; this record also included values that were outside the set limits. All patients in both study groups were treated according to Guidelines for the Management of Severe Traumatic Brain Injury, 3^rd^ Edition [11] and institutional standards. The management protocol included the following guidelines: ICP was maintained at < 20 mmHg, cerebral perfusion pressure (CPP) > 60 mmHg, Reductions in ICP were achieved by elevating the head of the bed >30°. If body temperature rose above 38°C, cooling blankets were used to restore and maintain body temperature between 36–38 °C. Other procedures included deeper sedation, drainage of cerebrospinal fluid, and maintaining of PaCO_2_ within 35– 40 mmHg, administration of 20% mannitol (0.25–1 g/kg), intermittent hyperventilation, and craniectomy. Low CPP was managed by administration of norepinephrine.

### Haemodynamic management

To assess hemodynamic status and to guide hemodynamic management the invasive hemodynamic monitoring was used. The Vigileo® monitor (Edwards Lifesciences, Irvine, CA, USA) or the The PiCCO® monitoring system (PULSION Medical Systems, Munich, Germany) was used. The choice of a particular monitor was at the discretion of attending physician. The cardiac index (CI) was maintained above 2.5 l · min · m-2 and was considered adequate if no signs of organ hypoperfusion were present. If the CI was considered low, adequate action was taken to increase it. If the patient was considered fluid responsive a bolus of fluids was given. The fluid responsiveness was assessed by stroke volume variation and a value of 12% was used as a threshold for administration of fluid bolus. If the CI was low and the patient was consider fluid unresponsive, the infusion of dobutamine was initiated. If hypotension with MAP below 65 mmHg and an SVV below 12% occurred, norepinephrine therapy was initiated.

Transfusion of packed red blood cells was used to maintain haemoglobin levels between 80–100 g/l.

### Mechanical ventilation

Protective ventilation strategies were used in both study groups. The tidal volume was set to 6-8ml/kg of ideal body weight, PEEP was set to 1cm H_2_O per 10kg of body weight. Further adjustments were made to keep airway plateau pressure below 30 cmH_2_O, arterial partial pressure of oxygen and CO_2_ in normal range.

### The ICP+PbtO_2_ group

In this group, the same protocol as in the ICP group was used augmented with therapeutic procedures to maintain PbtO_2_ > 20 mmHg. Management of the low PbtO_2_ included optimization of both ICP and CPP using the interventions noted above. To optimize oxygen delivery, the transfusion of packed red blood cells was administered to keep haemoglobin level at the upper end of the range, which was 100 g/l. In cases in which PbtO_2_ remained low despite optimization of ICP, CPP and haemoglobin level, the FiO_2_ was increased to maintain PbtO_2_ above 20 mmHg [12].

Patient demographic data as well as information on the type, mechanism and severity of the injury and hospital course were collected from electronic medical records and compared between study groups. Number of patients requiring transfusion of packed red blood cells and number of transfusion units were compared between study groups.

Clinical data (laboratory values, hemodynamic measurements, respiratory monitoring, length of mechanical ventilation, and measurements of ICP, CPP and PbtO_2_ were collected from both electronic and paper medical records. The means of all available values were calculated and compared between groups.

To assess clinical outcome of the patients the Glasgow Outcome Scale (GOS) was used. The values of GOS 30 days and 6 months after injury were compared between study groups. Overall outcome 30 days and 6 months after injury was dichotomised into favourable and unfavourable and compared between study groups. It was identified as favourable if the Glasgow Outcome Scale (GOS) score was 5 (good recovery, return to normal life) or 4 (moderate disability, although remaining independent in daily life). Unfavourable outcomes included GOS scores of 3 (severe disability, dependent in daily life), 2 (neurovegetative state), or 1 (death). The follow-up included survey findings from medical records or direct data collection via telephone.

Continuous variables are presented as mean ± standard deviation (SD). Categorical variables are described using tables of frequency. We used a two-tailed t-test for the comparison of continuous variables between the two groups. Categorical variables were compared between groups by χ^2^ analysis. *P*-values < 0.05 were used as a measure of statistical significance. We addressed missing data by re-analysing source data.

## Results

Seventy-seven patients were admitted to the Level I Trauma Centre for the treatment of severe TBI who fulfilled our inclusion criteria. Thirty-seven patients were managed via a protocol that included monitoring of both ICP and PbtO_2_ and included in the ICP+PbtO_2_ group while 40 patients were managed with ICP-guided monitoring alone and included in the ICP group. Patient demographics, mechanism of injury, type of injury, Injury Severity Score, need for vasopressors at admission, initial heart rate, initial mean arterial pressure, initial ICP, initial GCS, initial CT-based categories of injury, Marshall CT classification, and the need for surgery are summarized in Table 1. Characteristics of study groups were compared and there was no statistically significant difference identified. Twenty four (60.0%) of the patients in the ICP group received transfusion of packed red blood cells. The mean number of transfusion units administered was 3.8 ± 3.4. In the ICP+PbtO_2_ the packed red blood cells was administered to 21 (56.8%) of patients. In this group the mean number of transfusion units administered was 4.2 ± 2.6. The number of patients who received transfusion and the mean number of units transfuse didn’t differ significantly (*p* value 0.773 and 0.566 respectively). Cerebral monitoring data are outlined in Table 2.

**Table 1:** Characteristics of study population

Characteristics	-	Total (n=77)	ICP (n=40)	ICP+PbtO_2_ (n=37)	p
Age (years)	-	33.4 ± 11.4	31.6 ± 10.8	35.3 ± 11.7	0.144

Gender	M	60 (77.9%)	32 (80.0%)	28 (75.7%)	0.78
	F	17 (22.1%)	8 (20.0%)	9 (24.3%)	

Mechanism of injury	Fall	25 (32.5%)	12 (30.0%)	13 (35.1%)	0.875
	Motor vehicle accident	45 (58.4%)	24 (60.0%)	21 (56.8%)	
	Pedestrian–vehicle collision	7 (9.1%)	4 (10.0%)	3 (8.1%)	

Type of injury	Isolated TBI	23 (29.9%)	12 (30.0%)	11 (29.7%)	0.746
	Polytrauma	54 (70.1%)	28 (70.0%)	26 (70.3%)	

ISS	-	30.1 ± 7.0	31.6 ± 8.7	28.8 ± 6.7	0.193

Vasopressors at admission	YES	31 (40.3%)	16 (40.0%)	15 (40.5%)	0.961
	NO	46 (59.7%)	24 (60.0%)	22 (59.5%)	

MAP at admission	mmHg	82.9 ± 8.5	81.9 ± 9.8	83.1 ± 8.5	0.535

HR at admisson	beat/minute	78.3 ± 13.0	77.6 ± 10.9	78.3 ± 13.2	0.849

Days on vasopressor	-	6(6)	6(6)	6(6)	0.857

initial GCS	3	53 (68.9%)	30 (75.0%)	23 (62.2%)	0.19
	4	10 (12.9%)	2 (5.0%)	8 (21.6%)	
	5	10 (12.9%)	6 (15.0%)	4 (10.8%)	
	6	4 (5.3%)	2 (5.0%)	2 (5.4%)	
	7	0 (0.0%)	0 (0.0%)	0 (0.0%)	
	8	0 (0.0%)	0 (0.0%)	0 (0.0%)	

initial CT categories	EDH	17 (22.1%)	8 (20.0%)	9 (24.3%)	0.64
	SDH	45 (58.4%)	23 (57.5%)	22 (59.5%)	
	SAH	53 (68.8%)	26 (65.0%)	27 (73.0%)	
	ICH	36 (46.8%)	14 (35.0%)	22 (59.5%)	
	DAI	11 (14.3%)	7 (17.5%)	4 (10.8%)	

initial CT Marshall classification	1	0 (0.0%)	0 (0.0%)	0 (0.0%)	0.568
	2	32 (41.6%)	16 (40%)	16 (43.2%)	
	3	12 (15.6%)	7 (17.5%)	5 (13.6%)	
	4	4 (5.2%)	1 (2.5%)	3 (8.1%)	
	5	19 (24.7%)	12 (30.0%)	7 (18.9%)	
	6	10 (13.0%)	4 (10.0%)	6 (16.2%)	

initial ICP (mmHg)	-	18.1 ± 7.9	16.6 ± 6.7	19.7 ± 8.9	0.54

Operation	CTO	35 (45.5%)	14 (35.0%)	11 (29.7%)	0.98
	CE	9 (11.7%)	5 (12.5%)	4 (10.8%)	

Mann-Whitney U test for continuous data; Fisher´s exact test for categorical data. *CE* craniectomy, *CTO* craniotomy, *DAI* diffuse axonal injury, *EDH* epidural hematoma, *F* female, *GCS* Glasgow Coma Scale, *HR* heart rate, *ICH* intracerebral hematoma, *ISS* Injury Severity Score, *M* male, *MAP* mean arterial pressure*, SAH* subarachnoid haemorrhage, *SDH* subdural hematoma, *TBI* Traumatic Brain Injury

**Table 2:** Cerebral Monitoring Data

Cerebral monitoring	ICP	IPC+PbtO_2_	P
ICP (mmHg)	12.2 ± 2.8	10.1 ± 2.5	0.142
CPP (mmHg)	77.0 ± 4.3	76.5 ± 3.6	0.973
PaO_2_ (mmHg)	112.5 ± 7.5	140.3 ± 15.0	**0.045**
PaCO_2_ (mmHg)	37.5 ± 2.3	40.5 ± 3.8	0.789
PbtO_2_ (mmHg)	-	25.8 ± 4.0	-

Continuous data are presented as mean ± SD; Mann-Whitney U test for continuous data; *p*<0.05 as shown in bold. *CPP* cerebral perfusion pressure, *ICP* intracranial pressure

The mean ICP was lower in the patients who were managed with PbtO_2_ and ICP-guided monitoring compared with those who were monitored with ICP alone, although this difference did not achieve statistical significance. The mean arterial oxygen partial pressure was significantly higher in the group of patients managed with PbtO_2_ (112.5 *versus* 140.3 mmHg, *p* = 0.045), while the mean cerebral perfusion pressure and arterial partial pressure of CO_2_ were similar between the two groups.

The median GOS scores at 30 days after TBI were similar in both groups of patients; better outcomes overall were observed at 6 months post-TBI. At this later time point, the median GOS score was significantly higher (*p* = 0.013) in the group that was managed with the PbtO_2_ monitoring protocol. Mortality rates recorded at 6 months for patients managed with ICP alone *versus* those managed with ICP and PbtO_2_ were 20% and 10.8%, respectively, although these differences did not achieve statistical significance. Except for one patient in the PbtO_2_ group who died secondary to the progression of respiratory insufficiency caused by severe lung injury, a direct or indirect association between TBI and cause of death was found in all remaining patients. Favourable outcomes (GOS scores of 4 or 5) were observed more frequently in the patient group that was monitored using the PbtO_2_ protocol (*p* = 0.029). We didn´t found any difference in incidence of favourable outcomes and mechanism nor type of injury. Details of this analysis are shown in Table 3.

**Table 3:** Clinical outcome data

Outcome	ICP	ICP+PbtO_2_	p
Mechanical ventilation (days)	11.5 (9)	10 (6.5)	0.502

ICU LOS	17 (14)	15.5 (10)	0.857

Hospital LOS	29 (11)	24 (12)	0.246

GOS score 30 days after injury	3 (1,5)	3(1,5)	0.107

GOS score 6 months after injury	3(2)	4(2)	**0.013**

180-days mortality	8 (20.0%)	3 (10.8%)	0.335
Favourable Outcomes (GOS 4 or 5) 30 days after TBI	10 (25.0%)	14 (37.8%)	0.224
Mechanism of injury			
Fall	3 (7.5%)	6 (16.2%)	0.809
Motor vehicle accident	6 (15.0%)	7 (18.9%)	
Pedestrian–vehicle collision	1 (2.5%)	1 (2.7%)	
Type of injury			
Isolated TBI	1 (2.5.%)	5 (13.5%)	0.151
Polytrauma	9 (22.5%)	9 (24.3%)	

Favourable Outcomes (GOS 4 or 5) 6 months after TBI	16 (40.0%)	24 (64.9%)	**0.029**
Mechanism of injury			
Fall	4 (10.0%)	9 (24.3%)	0.700
Motor vehicle accident	11 (27.5%)	14 (37.8%)	
Pedestrian–vehicle collision	1 (2.5%)	1 (2.7%)	
Type of injury			
Isolated TBI	4 (10.0%)	7 (18.9%)	0.772
Polytruama	12 (30.0%)	17 (45.9%)	

Continuous data are presented as median (IQR); Mann-Whitney U test for continuous data; Fisher´s exact test for categorical data; *p*< 0.05 are shown in bold. *GOS* Glasgow Outcome Scale, *ICU* Intensive Care Unit, *LOS* Length of Stay, *TBI* Traumatic Brain Injury

## Discussion

The management of TBI patients focuses on the prevention of secondary brain injury. Brain tissue hypoxia is one of the major mechanisms underlying secondary brain injury and has been associated with unfavourable outcomes. Therefore, strategies and management based on monitoring of brain tissue oxygenation may have a positive impact on clinical outcomes in TBI patients. Results from several studies in which severe TBI was managed by monitoring PbtO_2_ revealed trends towards lower mortality rates and more favourable outcomes [9]. However, other studies found that PbtO_2_ monitoring resulted in no positive effects [13, 14]. Recent meta-analysis of tree randomised controlled trials analysed data from 214 patients and found positive effect on mortality when PbtO_2_ guided management was used. There was no positive effect on neurological outcome, however the certainty of evidence was very low [15]. Current guidelines for the management of severe TBI discussed the contradictory nature of these findings [2]. In contrast to the previous guidelines, PbtO_2_ monitoring is no longer recommended [11]. However, protocols were recently published that can be used to direct the management of TBI patients using both ICP and PbtO_2_ monitors [16]. Of significant interest, PbtO_2_ monitoring is included in the current guidelines for the Management of Paediatric Severe Traumatic Brain Injury [17].

In the present study, we performed a retrospective analysis of outcomes in TBI patients who were managed with both ICP and PbtO_2_ monitoring or with ICP monitoring alone.

The impact of PbtO_2_ monitoring on mortality reported in several previous studies remained unclear and unconvincing [10, 12, 13, 14 18]. In our study, although overall mortality was lower in the group of patients managed with PbtO_2_ monitoring (10.8% *versus* 20% for the group managed with ICP-guided protocols alone), this finding did not achieve statistical significance. We also recognize that the studies that reported significantly lower mortality rates among those managed with PbtO_2_ monitoring used historical controls [13, 14]. Reduced mortality rates associated with ICP and PbtO_2_ monitoring were reported in several studies, although, similar to our findings, none of the differences achieved statistical significance [8, 9, 10 19]. While the mortality rates in our study were lower than those reported by Green et al. [10], these researchers evaluated mortality only at the time of hospital discharge. By contrast, our study included an analysis of mortality up to and including 180 days after the acute injury. We note that our dataset covered a period that was 10 years later than that evaluated by Green et al. [10]; some of the differences may relate to overall progress made in general patient care since that time. We also recognize that our patient cohort was younger than that enrolled in this earlier study.

Our results indicate that patients managed with ICP and PbtO_2_ monitoring have more favourable outcomes than patients managed with ICP monitoring alone. Specifically, our findings revealed a statistically significant difference in GOS scores in these patient groups at 6 months after TBI. A trend toward improved outcomes was found one month after TBI, but the difference was not statistically significant. Our results suggest that recovery after TBI is a long process. Similar results were reported in other studies [8, 20, 21].

Favourable outcomes are an important indicator of treatment success [22, 23, 24]. Favourable outcomes may be defined as scores of 4–5 on the GOS or 5–8 on the Glasgow Outcome Scale-Extended (GOS-E) [23, 24, 25]. Our results included a higher fraction of patients with favourable outcomes compared to results reported by Okonkwo et al. [9]. Specifically, our findings included a higher rate of positive outcomes among those managed with ICP and PbtO_2_ (64% *versus* 41%) and ICP alone (40% *versus* 31%). Similarly, Lin et al. [8] reported favourable outcomes in fewer than 30% of the patients in both groups at one month after injury. Collectively, these results suggest that patients managed by ICP and PbtO_2_ monitoring are more likely to exhibit favourable outcomes as their neurological conditions develop and improve over time. Thus, patient outcomes might be evaluated over longer periods to obtain a true assessment of the impact of these management strategies. We didnť find any difference in incidence of favourable outcomes regarding mechanism and type of injury, however numbers of patients in assessed categories were small which may have influenced the results.

This study has several limitations. First, this study was designed as a retrospective analysis and is associated with the limitations inherent in all studies of this type. Second, this was a single-centre study that included a limited number of patients and thus lacks external validity. Third, the positioning of the PbtO_2_ and/or ICP probes was not standardized; the site of insertion was determined independently by each of the attending neurosurgeons. Fourth, the retrospective design and relatively small sample size may be a source of selection and performance biases. Finally, the GOS evaluation was challenging, especially identifying patients in the GOS category 3. This category was considered to be an adverse outcome; however, it was not always easy to determine whether a patient’s disability was the direct result of the TBI as opposed to associated injuries.

Several studies reported positive trends with respect to mortality and outcomes of TBI patients who were managed by PbtO_2_ monitoring, while others did not [9, 12, 18 ]. Therefore, the role of PbtO_2_ in the management of TBI patients and its impact on patient outcomes remains unresolved. Results of our study suggest that management of adult TBI patients using PbtO_2_ monitoring might have a positive impact on clinical outcomes. The BOOST3 trial (ClinicalTrials.gov identifier: NCT03754114) which is currently ongoing (anticipated completion date of July 2023), aims to evaluate the impact of multimodal ICP and PbtO2 treatment on neurological outcomes.

## Conclusions

Appropriate monitoring and management of TBI to prevent secondary brain injury remain challenging. The role of PbtO_2_-based guidance in the management of TBI remains an unresolved but promising subject for future research. Results of well-designed randomized controlled trials will be needed to clarify the role of PbtO_2_ monitoring in TBI patients.
